# Stability in and Correlation between Factors Influencing Genetic Quality of Seed Lots in Seed Orchard of *Pinus tabuliformis* Carr. over a 12-Year Span

**DOI:** 10.1371/journal.pone.0023544

**Published:** 2011-08-24

**Authors:** Wei Li, Xiaoru Wang, Yue Li

**Affiliations:** 1 National Engineering Laboratory for Forest Tree Breeding, Key Laboratory for Genetics and Breeding of Forest Trees and Ornamental Plants of Ministry of Education, Beijing Forestry University, Beijing, People's Republic of China; 2 State Key Laboratory of Systematic and Evolutionary Botany, Institute of Botany, Chinese Academy of Sciences, Beijing, People's Republic of China; University of California, United States of America

## Abstract

Coniferous seed orchards require a long period from initial seed harvest to stable seed production. Differential reproductive success and asynchrony are among the main factors for orchard crops year-to-year variation in terms of parental gametic contribution and ultimately the genetic gain. It is fundamental in both making predictions about the genetic composition of the seed crop and decisions about orchard roguing and improved seed orchard establishment. In this paper, a primary Chinese pine seed orchard with 49 clones is investigated for stability, variation and correlation analysis of factors which influence genetic quality of the seed lots from initial seed harvest to the stable seed production over a 12 years span. Results indicated that the reproductive synchrony index of pollen shedding has shown to be higher than that of the strobili receptivity, and both can be drastically influenced by the ambient climate factors. Reproductive synchrony index of the clones has certain relative stability and it could be used as an indication of the seed orchard status during maturity stage; clones in the studied orchard have shown extreme differences in terms of the gametic and genetic contribution to the seed crop at the orchard's early production phase specifically when they severe as either female or male parents. Those differences are closely related to clonal sex tendency at the time of orchard's initial reproduction. Clonal gamete contribution as male and female parent often has a negative correlation. Clone utilization as pollen, seed or both pollen and seed donors should consider the role it would play in the seed crop; due to numerous factors influencing on the mating system in seed orchards, clonal genetic contribution as male parent is uncertain, and it has major influence on the genetic composition in the seed orchard during the initial reproductive and seed production phase.

## Introduction

Seed orchards are the most common means of making available a stable supply of genetically improved seed and it constitutes an important component in most coniferous species improvement programs in the world [1]. Genetic gain of seed orchards' crops depends on the section differential between the orchards' parental populations and that of unselected seed sources as well as orchards' parental population actual gamete contribution to the harvested seed crops [2]. The attainment of balanced gametic contribution from orchards' parents is hardly observed due to many factors including differential male and female reproductive success and asynchrony as well as the degree of selfing and pollen introgression from outside unselected sources [3] [4]. Moreover coniferous trees have a long lifespan, thus seed orchards require a long period from the initial establishment until reproductive maturity and the steady production of a stable seed yield. Differential reproductive success and asynchrony are among the main factors for orchard crops year-to-year variation in terms of parental gametic contribution and ultimately the genetic gain [5]. Therefore, the time span from initial seed harvest to stable seed production shows substantial parental gametic contribution instability and is the subject of intense research [6] [7].

Chinese pine (*Pinus tabuliformis* Carr.) is an important native coniferous tree species to northern China and is naturally distributed across 14 northern provinces and autonomous regions, with a land cover of close to 3 million km^2^ [8]. Because of the important ecological and economy status of Chinese pine as indigenous conifer tree species in northern China, a tree improvement program was initiated in the 1970 s that was followed by clonal seed orchard establishment [9]. Many of the basic aspects of species' genetic improvement such as grafting technology, pollen dynamics and parental gamete contribution have been comprehensively and systematically studied [10] [11]. Currently, it is inadequate in addressing the flowering synchronization between clones, annual relevant gamete contribution, stability of genetic gain of the seed lots, factors influencing the genetic structure of the seed lots and relationships between factors influencing the genetic quality of seed lots during the period of from initial seed harvest to the stable seed production in the primary Chinese pine seed orchard.

In this paper, we evaluate clonal reproductive success and synchrony among 49 Chinese pine clones growing in a seed located in Xingcheng City (Liaoning Province, China). In particular, we investigate the flowering phases, quantity of female and male flowers as well as the average number of full seeds in each cone of selected clones and their impact on seed crops breeding value over a period of 12 years. Results are expected to provide the theoretical foundation for the seed orchard management, genetic composition prediction and advanced seed orchard establishment.

## Materials and Methods

### Ethics statement

This research only involved in coniferous trees and general station of forest seedling management of Liaoning Province has agreed our observational and field studies. We have published several other papers with the same material.

### Materials

This study was carried out in a primary clonal Chinese pine seed orchard which located in Xingcheng City Liaoning Province of China (40°44′N, 120°34′E, 100 m above sea level). The seed orchard is composed of 49 clones planted at 7 m×7 m fixed block design. Clones were grafted in 1974 using two-year rootstock and planted in 1975 [12]. Samples and investigation were carried out in the 10th, 13th, 18th, and 21st years after grafting spanning 12 years covering the time from initial flowering to stable seed production.

### Methods

Four ramets were randomly selected to represent each clone. Within each ramet, 10 female strobili were marked at the central part of sunny side of the selected plants representing the female component. Additionally the male component was represented by 10 male strobili branches located in the short middle branches on the shady side where most male strobili were positioned. Efforts were made to reach a level of sampling consistency throughout the study period [13].

In the years of observation, the marked strobili, male and female, were monitored at fixed time (noon) throughout the reproductive stage of each clone, and based on this the number of female and male strobili in each sample plant was calculated. At the same time, the average number of filled seeds in a sample of 30 seed-cones was determined (note: this was done the year following pollination as pine cones require 2 years to reach maturity).

Flowering process was divided 4 stages which have been described in other conifer tree species. The female stages were described by Matziris [14] and Codesido *et al*. [15]: stage 1, the female bud is increasing in size, becomes cylindrical, but is still completely covered by the bud scales (0% female receptivity); stage 2, the apex of the enlarged cylindrical bud is opened and the first ovuliferous scales appear (20% female receptivity); stage 3, the scales of the female conelet are gradually separated and almost form right angles with the axis of the conelet (100%), and stage 4, the ovuliferous scales increase in size and thickness (0% female receptivity). The male stages were described as follows [15] [16]: stage 1, the round brown strobili are covered by the bud scales (0% pollen shedding); stage 2, the male strobili burst through the bud scales and elongate (0% pollen shedding); stage 3, the yellow strobili start shedding their pollen (100% pollen shedding) and stage 4, end of pollen shedding. The male strobili wither and fall down (0% pollen shedding).

### Date analysis

Index of measuring phonological overlap (*PO_ij_*) proposed by Askew and Blush [17] was used for quantifying the degree of reproductive synchronization. When mating pairs have exactly same flowering phases, *PO_ij_* value will reach the maximum 1. Conversely, when the flowering phases do not overlap, *PO_ij_* value equals to minimum 0. When the flowering phases partially overlapped, *PO_ij_* value will range between 0 and 1. If *i* = *j*, *PO_ij_* means selfing flowering synchronization within the same clone.

According to the method described by El-Kassaby and Askew [18] and Askew [19], the relative gametic reproduction of a specific clone as female or male was calculated by the ratio of the average quantity of female or male strobili in all sampled ramets to the total number overall strobili average of all clones in the seed orchard. Clonal breeding value was determined from progeny testing and gain values of tree height at age 20 were considered in the following analyses.

Parental gamete contribution to the seed lots of seed orchard was estimated with the following method:
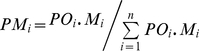
(1)

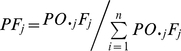
(2)

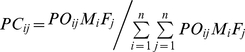
(3)in which, *PM_i_* and *PF_j_* are the gamete ratio of the clones *i* and *j* as male and female parent respectively in the seed orchard; *PC_ij_* is the gamete ratio of the clone *i* and *j* as mating pair in seed orchard; *PO_i_*. and *PO_.j_* are the average flowering synchronization indices of the clone *i* and *j* as male and female parent respectively in the seed orchard; *M_i_* and *F_j_* are the pollen loading and ovules number of the clone *i* and *j* in the seed orchard.

For a clonal seed orchard with *t* clones, the relative genetic contribution of the clone *j* as female parent (*GA_j_*) can be formulated as following:
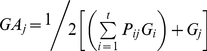
(4)

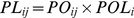
(5)

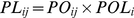
(6)in which, *G_i_* and *G_j_* represent the relative breeding value of the clones *i* and *j*, respectively; *P_ij_* is the seed ratio in the strobilus of clone *j* which pollinated by clone *i*; *PL_ij_* is calculated with the relative male flowers quantity (*POL_i_*) and flowering synchronization index (*PO_ij_*).

Correlation analysis was estimated as Pearson correlation (*r*) and was used to determine the extent of relationship between all the indexes. Special analysis software developed by Huang and Chen [20] was used for the date processing.

## Results

### Variation in clonal reproductive synchrony

**Figure 1:**
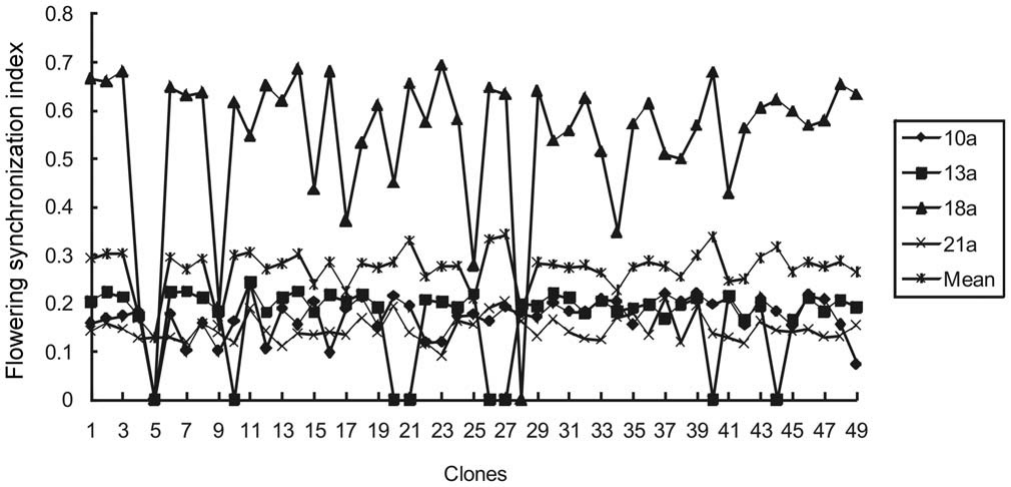
Clonal flowering synchronization as female in *Pinus tabuliformis* seed orchard.

**Figure 2:**
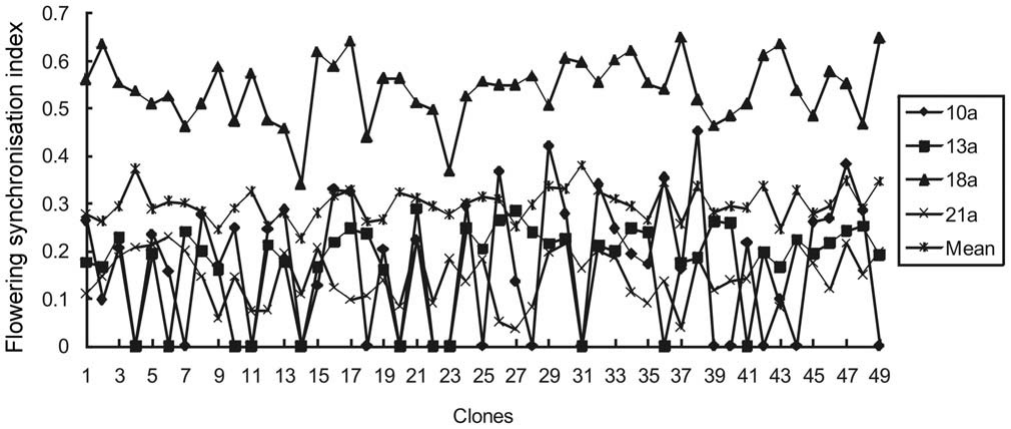
Clonal flowering synchronization as male in *Pinus tabuliformis* seed orchard.

Reprodductive synchrony index among clones as female parents ranged from 0.146 to 0.563 with average of 0.249. Across observation years, reproductive synchrony index as female parent was below 0.2; however, the 18th year produced an index of 0.563 and that was mainly caused by the damp cool weather that caused prolonged receptivity period (Fig. 1). Male reproductive synchrony index ranged from 0.145 to 0.297. Except for the abnormal data of the 21st year, all values were over 0.2 and it was higher than that of female parents (Fig. 2).

Correlation analysis shows that the flowering synchronization index of male parent in the 10th year has a significant positive correlation with that in the 13th and 21st years, significant negative correlation with that in the 18th year, and no significant correlation with the observations in other years. Conversely, the flowering synchronization index of female parents has a positive correlation between most of the observed years and some correlations reach to significance level. The flowering synchronization index of selfing in the 10th year has a significant correlation with that in the 13th year, and those in the other years have no significant correlation. The flowering synchronism index of selfing has a positive correlation with that of as male parent, and reachs to significance level in the 10th and 13th years. The study showed that reproductive synchrony index of the clones has certain relative stability and thus could be used as an indication of the seed orchard status during maturity (Table 1).

**Table 1:** Within clone male and female flowering synchrony in *Pinus tabulaeformis* seed orchard.

	M10	F10	MF10	M13	F13	MF13	M18	F18	MF18	M21	F21
F10	0.01										
MF10	0.79**	0.15									
M13	0.42*	0.05	0.28								
F13	0.07	0.10	−0.02	0.10							
MF13	0.22	0.09	0.43*	0.55**	0.04						
M18	−0.50*	0.36	−0.34	−0.19	0.04	−0.23					
F18	0.18	−0.11	−0.06	0.07	0.34	0.04	−0.45*				
MF18	−0.12	0.02	−0.15	−0.04	0.01	0.12	0.11	0.63**			
M21	0.35*	−0.05	0.03	−0.08	−0.07	0.02	−0.10	0.08	0.34		
F21	−0.05	0.38*	0.21	−0.10	0.94**	0.21	0.34*	−0.39**	0.56	−0.05	
MF21	0.44**	0.13	0.32	−0.12	0.75**	0.24	0.14	0.54**	0.14	−0.27	0.56**

Note: M10 means clonal gametic contribution as male in the 10th year after clone grafting in the seed orchard; F10 means clonal gametic contribution as female in the 10th year after clone grafting in the seed orchard; MF10 means clonal gametic contribution as both male and female in the 10th year after clone grafting in the seed orchard; the same with others.

*represents significant at 0.05 level;

**represents significant at 0.01 level; similar comments apply to below tables.

### Clonal gametic contribution variation

**Figure 3:**
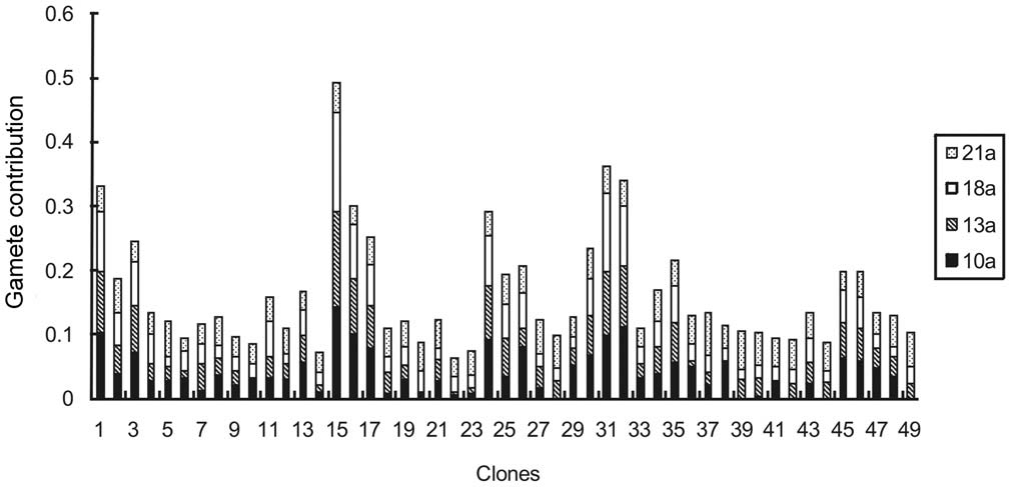
Gametic contribution of clones in *Pinus tabuliformis* seed orchard.

**Figure 4:**
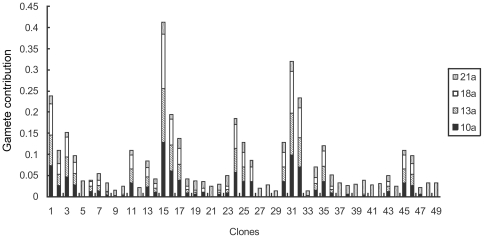
Clonal gametic contribution as female in *Pinus tabuliformis* seed orchard.

**Figure 5:**
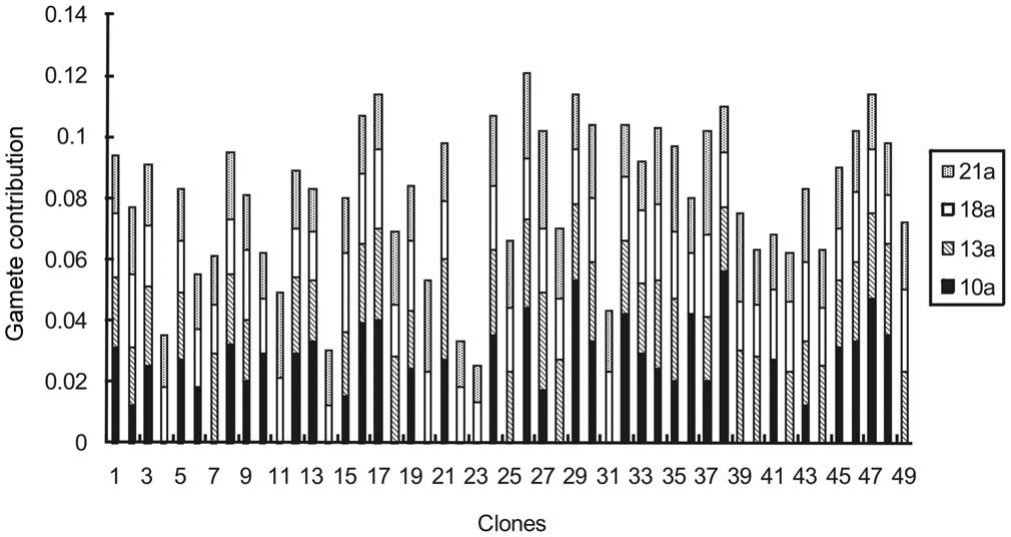
Clonal gametic contribution as male in *Pinus tabuliformis* seed orchard.

The average gamete contribution of each clone served as both male and female parents is about 0.04 across study years. At the initial stage of seed production, great variations occurred between the clones and the variation coefficient reached 55.20% among the annual average gamete contributions of all the clones. For the studied 49 clones, clone 15 had the largest gamete contribution with an annual average of about 7.8 times greater than that of clone 22 (the smallest contributer) (Fig. 3). But the gamete contribution is also very different when the clones served as single male and female parent (Fig. 4 and 5). Variation of the gamete contribution among the clones as female parent is much big than that of male parent across study years and the variation coefficient in the annual female gamete contribution is greater than 1, indicating that there is a substantial difference among clonal gametic contribution specifically at the initial seed production stage (Table 2).

**Table 2:** Gametic contribution related values in *Pinus tabuliformis* seed orchard.

Clone	Breedingvalue	Flowering synchronization			Gamete contribution	Genetic contribution	Clone	Breedingvalue	Flowering synchronization			Gamete contribution	Genetic contribution
		Father	Mother	Both					Father	Mother	Both		
1	0.16	0.277	0.268	0.0414	0.0414	0.0132	26	0.13	0.306	0.332	0.0261	0.0261	0.0068
2	0.15	0.285	0.272	0.0233	0.0233	0.0070	27	0.12	0.260	0.300	0.0152	0.0152	0.0036
3	0.14	0.294	0.272	0.0306	0.0306	0.0086	28	0.14	0.246	0.163	0.0122	0.0122	0.0034
4	0.06	0.372	0.167	0.0166	0.0166	0.0020	29	0.08	0.335	0.252	0.0159	0.0158	0.0025
5	0.23	0.294	0.128	0.0151	0.0151	0.0069	30	0.10	0.328	0.259	0.0292	0.0292	0.0059
6	0.18	0.304	0.274	0.0119	0.0119	0.0043	31	0.15	0.380	0.250	0.0453	0.0453	0.0136
7	0.22	0.301	0.229	0.0144	0.0144	0.0064	32	0.10	0.327	0.251	0.0425	0.0425	0.0085
8	0.13	0.283	0.256	0.0160	0.0160	0.0042	33	0.14	0.312	0.243	0.0135	0.0135	0.0038
9	0.12	0.234	0.146	0.0121	0.0121	0.0029	34	0.17	0.294	0.208	0.0147	0.0147	0.0072
10	0.17	0.269	0.298	0.0108	0.0108	0.0037	35	0.09	0.280	0.238	0.0271	0.0271	0.0049
11	0.08	0.324	0.273	0.0197	0.0197	0.0032	36	0.21	0.343	0.254	0.0163	0.0163	0.0068
12	0.19	0.253	0.236	0.0137	0.0137	0.0052	37	0.14	0.274	0.252	0.0168	0.0168	0.0047
13	0.16	0.280	0.259	0.0208	0.0208	0.0067	38	0.13	0.336	0.232	0.0173	0.0173	0.0037
14	0.13	0.226	0.266	0.0089	0.0089	0.0023	39	0.18	0.281	0.281	0.0132	0.0132	0.0047
15	0.15	0.280	0.221	0.0616	0.0616	0.0185	40	0.11	0.294	0.337	0.0128	0.0128	0.0028
16	0.10	0.308	0.263	0.0376	0.0376	0.0075	41	0.12	0.290	0.228	0.0118	0.0118	0.0028
17	0.14	0.305	0.216	0.0313	0.0313	0.0088	42	0.16	0.336	0.217	0.0114	0.0114	0.0037
18	0.12	0.261	0.263	0.0135	0.0135	0.0033	43	0.19	0.259	0.256	0.0165	0.0165	0.0063
19	0.19	0.273	0.236	0.0151	0.0150	0.0057	44	0.17	0.327	0.315	0.0109	0.0109	0.0037
20	0.04	0.267	0.261	0.0107	0.0107	0.0009	45	0.09	0.300	0.224	0.0249	0.0249	0.0045
21	0.16	0.295	0.329	0.0154	0.0154	0.0049	46	0.07	0.276	0.262	0.0246	0.0246	0.0035
22	0.19	0.294	0.254	0.0079	0.0079	0.0030	47	0.12	0.351	0.252	0.0167	0.0167	0.0040
23	0.20	0.276	0.248	0.0092	0.0092	0.0037	48	0.23	0.311	0.261	0.0162	0.0162	0.0075
24	0.12	0.301	0.245	0.0363	0.0363	0.0087	49	0.20	0.346	0.263	0.0129	0.0129	0.0052
25	0.13	0.314	0.195	0.0241	0.0241	0.0063	mean	0.14	0.297	0.249	0.0200	0.0200	0.0055

Except for the 21st year, gametic contribution of each clone as male, female, and as all parent in the 10th, 13th, and 18th years has significant or highly significant correlation with each other. Cloanal gametic contribution as male and female parents in the 10th, 13th and 21st years produced correlations higher than 0.9, again indicative of relative stability over the study period (Table 3).

**Table 3:** Clonal gametic contribution correlation in *Pinus tabulaeformis* seed orchard.

	M10	M13	M18	M21	F10	F13	F18	F21	MF10	MF13	MF18
**M13**	0.81**										
**M18**	0.51**	0.59**									
**M21**	−0.34	−0.24	−0.17								
**F10**	−0.09	−0.12	−0.19	−0.13							
**F13**	−0.21	−0.28	−0.11	−0.11	0.61**						
**F18**	−0.33	−0.36*	−0.52**	0.17	0.27	0.39*					
**F21**	0.31	0.21	0.17	−0.01	0.13	−0.33	−0.05				
**MF10**	−0.38*	−0.44*	−0.37*	−0.01	0.60**	0.36*	0.57**	−0.05			
**MF13**	−0.28	−0.34	−0.20	−0.18	0.69**	0.51**	0.54**	0.00	0.90**		
**MF18**	−0.30	−0.37*	−0.20	−0.20	0.65**	0.50**	0.50**	0.00	0.91**	0.97**	
**MF21**	0.33	0.33	0.36*	−0.07	0.02	−0.29	−0.16	0.83**	−0.16	−.06	−0.01

### Clonal variation in genetic contribution

**Figure 6:**
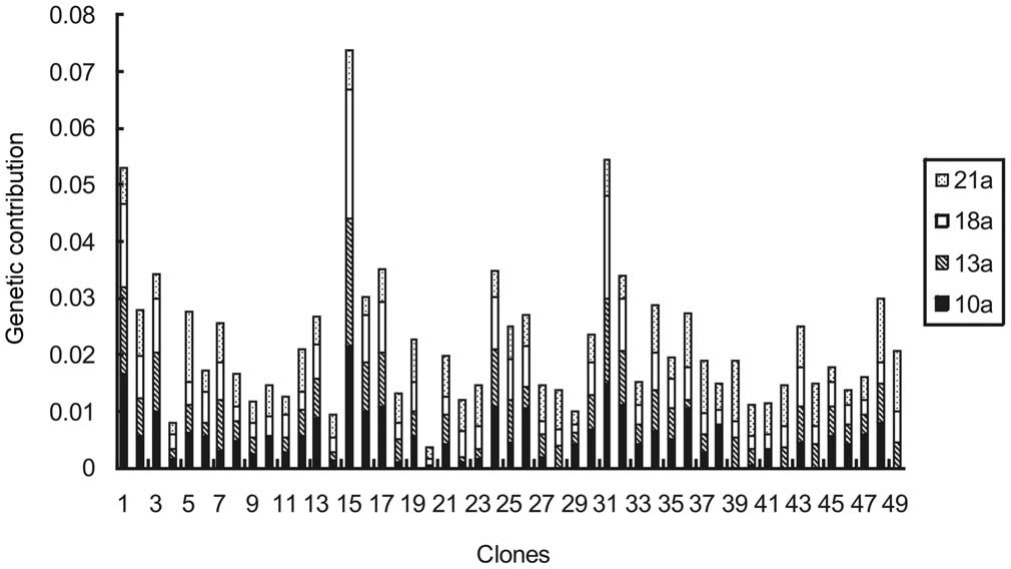
Genetic contribution of clones in *Pinus tabuliformis* seed orchard.

**Figure 7:**
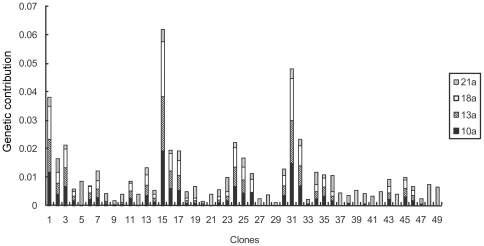
Clonal genetic contribution as female in *Pinus tabuliformis* seed orchard.

**Figure 8:**
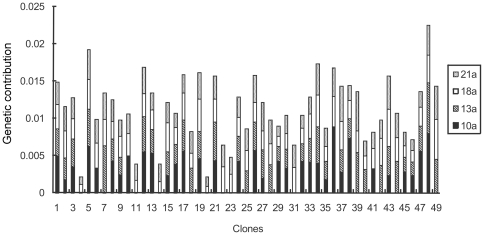
Clonal genetic contribution as male in *Pinus tabuliformis* seed orchard.

There is a significant difference in the estimated genetic contribution of the clones as male, female, and as parents to the orchard's zygote pool (Fig. 6, 7 and 8). At the initial reproductive stage, some clones failed to produce female or male strobili, so they did not contribute genetically to the seed crops, whereas other clones had higher than expected genetic contribution (e.g., clones 1, 15, and 24 as female parents) (Fig. 7 and 8). In the 21st year, all clones contributed to the zygote pool, especially those with high breeding values, thus increasing the seed crop's estimated genetic gain and reflecting the magnitude of genetic gain fluctuation over years (Fig. 6).

In the study years, the average genetic contribution of all the clones was 0.005541, and the variation coefficient reached 57.48%. The average estimated genetic contribution of each clones at different year substantially varied with clones 15 and 20 showing the highest and lowest average genetic contribution, respectively, with as high as 21 fold difference between them (Fig. 6). It demonstrates clonal effect on gametic contribution and subsequently the expected genetic gain. This information could serve as the bases for the seed orchard genetic thinning through the removal of low contributors.

### Factors influencing clonal genetic contribution

Parameters related to the gamete contribution of clones such as (flowering synchronization index, gamete contribution, quantity of female and male flowers and number of full seeds per cone) were estimated (Table 2). According to correlation analyses (Table 4), the genetic contribution of each clone has some degrees of correlation with its clonal breeding value. This indicates that these are the most important factors influencing the genetic contribution of each clone.

**Table 4:** Correlation between clonal gametic contribution and its related parameters in *Pinus tabuliformis* seed orchard.

Correlation efficience	Breedingvaule	Flowering synchronization	Gamete contribution	Number of female flowers	Number of male flowers	Full seeds per cones
Genetic Contribution	0.2666*	0.8589**	0.8589**	0.5840**	−0.3532*	0.6280**
Reliability	0.9560	0.9999	0.9999	0.9999	0.9671	0.9999

## Discussion

By analysis of reproductive synchrony and its annual stability across clones in a seed orchard from initial seed harvest to stable seed production, clonal female and male contribution can be effectively estimated. Based on this, the gamete and genetic contribution of clones to the seed crop can be calculated. Finally, with generated information, genetic thinning, clonal management measures can be adopted to adjust the genetic composition of orchard pool and to improve the level of genetic gain of the resulting seed crops. Results of this study show that the reproductive synchrony among orchard's clones has some variation among the studied years. The reproductive synchrony index of pollen release has shown to be slightly higher than that of the female receptivity; however, both can be drastically influenced by the ambient climate factors resulting in some changes. For example, the cool and damp weather during the 18th year significantly improved the reproductive synchrony in the studied orchard due to the extended receptivity period. This indirectly indicates that the use of techniques such as those affecting ambient conditions such as bloom delay can effectively alter reproductive synchrony. The reproductive synchrony index observed over short periods (i.e., few years) cannot reflect the relative difference among clones and thus longer observation periods conducted over several years are needed to effectively evaluate the differences in reproductive synchrony among orchard's clones.

Parental contribution to the seed orchard gametic pool actually means the contribution to the seed crop. Askew and Blush [17] and El-Kassaby and Askew [18] proposed a formula to calculate parental genetic contribution that focuses on the combination probability of opposite genders gametes from the different parents. In the present study, we used the relative gamete yield to estimate the gametic and genetic contribution of each clone as male, female and as parents. Clones in the study orchard have shown extreme differences in terms of the gametic and genetic contribution to the seed crop at the orchard's early production phase. Although it can reflect the relative importance of different clones as male, female and as parents to the genetic composition of the seed crop, the estimation of gametic and genetic contribution of the parents is based on ideal assumptions and actual estimation of genetic contribution of the male parent is uncertain. It subjects to many factors which are influencing the orchard's mating system [21] [22] [23]. Hence, genetic contribution to the seed crop can be finally realized based on the contribution of the female parent. In actual, clonal gamete contribution as male and female often has a negative correlation [23], so the utilization of a clone as pollen, seed or pollen and seed donors should consider the role it should play in the seed crop.

When the clone serves as different parents (i.e., male and/or female), gametic contribution to the seed orchard has a close positive correlation between the observed years. This indicates the presence of certain stability in clonal gametic contribution across the orchard's productive life span. Because genetic contribution is the function of gametic contribution and breeding value, so the genetic contribution of clones is also relatively stable and it can be used to evaluate the genetic composition of seed crops. However, great variation appears in clonal gametic and genetic contribution specifically when they severe as either female or male parents across the study years and those variations are closely related to clonal gender tendency and the features during the orchard's initial reproduction [12]. So genetic composition analysis of seed crops produced from any seed orchard should take the whole clone as the analytic unit of gametic and genetic contribution.

Stability of clonal gametic contribution across years is affected by numerous factors. Generally, gametic contribution of a clone is related to reproductive synchrony between opposite gender, the quantity of female and male strobili production, and based on assumption of no significant difference in the number of valid gametes produced by the female and male strobili (i.e., relationship between reproductive energy and reproductive success) [24]. Moreover, clone arrangement, non-random mating among clones and climate factors may also influence the estimation of gametic contribution, which it is very difficult to reliably estimate the number of viable gametes in the female stobili. Thus, the current study accepts the assumption and replaces the difference between viable female gametes in the clone with the average number of filled seeds produced. Although the estimation result has some variation with that of the actual gametic contribution, it is more convenient operationnally, and basically meets the requirements for seed orchard management. This study has shown that as the number of female and male strobili as well as the average number of filled seeds per cone, have great variances among clones and this variation is associated with relatively high stability during the observed years, and similarly variance in the reproductive synchrony between opposite gender also has its stability. Therefore, gametic contribution of Chinese pine clones in seed orchard demonstrates relatively high stability at the orchard's early reproductive phase. The genetic contribution of clones is the function of gametic contribution and breeding value, while the breeding value of the clone is relatively independent from its gametic contribution. For example, clones 5, 4, 23, 36, 48, and 50 possess a relative high breeding value (>0.20), but have lower than the average relative gametic contribution. Among clones with a relative higher gametic contribution, 9 clones have breeding values higher than the average, whereas 7 clones have lower than the average. This highlights the relative importance of different clones in the genetic gain of seed crops and the yield of seeds from seed orchard. Genetic contribution comprehensively demonstrates those two values of the clone.

In summary, due to influence of numerous factors on the mating system in seed orchards, genetic contribution of the Chinese pine clone as male parent is uncertain, genetic contribution of the male gamete from a clone has the greatest influence on the genetic composition of the seed orchard during the initial reproductive and seed production phase. Clones with a low breeding value and high contribution of male gametes may significantly reduce the genetic quality of seeds and this can be treated by routine genetic thinning or removal of male strobili carrying branches. Genetic gain from the genetic contribution of female parent to the seed crops has more realistic meanings. Improving yield of female strobili of a clone, which has low female gametic contribution and high breeding values, is an important and effective measure to enhance production and genetic quality of the seed crops. Improved Chinese pine seed orchard can be rebuilt through backward selection based on the general and special combining ability test, and the optimal genetic gain of seed crops from the seed orchard can be reached by optimization with value of general and special combining ability of the clones, reproductive synchrony of opposite genders, male and female gamete contribution to the zygote poll of the seed orchard. Consequently, genetic structure and clone combination can be designed in an improved seed orchard in which clones have the most effective genetic contribution to the seed lots.
